# Association between lipid profiles and presence of carotid plaque

**DOI:** 10.1038/s41598-019-54285-w

**Published:** 2019-11-29

**Authors:** Yanhua Liu, Yongjian Zhu, Wenrui Jia, Dan Sun, Li Zhao, Chen Zhang, Cuicui Wang, Gaiyun Chen, Sanxian Fu, Yacong Bo, Yurong Xing

**Affiliations:** 1 Department of Nutrition, the First Affiliated Hospital of Zhengzhou University, Zhengzhou, 450052 China; 2 Department of Cardiology, the First Affiliated Hospital of Zhengzhou University, Zhengzhou, 450052 China; 3 Depatment of Physical Center, the First Affiliated Hospital of Zhengzhou University, Zhengzhou, 450052 China

**Keywords:** Cardiovascular diseases, Risk factors

## Abstract

It is indicated that lipids profiles are associated with carotid plaque and Atherosclerosis. However, studies about the relationship between serum lipid profiles and carotid plaque composition in Chinese Population is limited. We conducted a cross-sectional study among 3,214 participants between January 2015 and December 2017 in China, to investigate the association between various lipid profiles and the prevalence of carotid plaque. Logistic regression model was used to investigate the association between plasma lipid profiles and odds of carotid plaque. Analysis of covariance (ANCOVA) was used to compare the mean plasma lipid profiles among different number and composition of carotid artery plaques. HDL-C, Non-HDL-C levels, TC/HDL-C, LDL-C/HDL-C were significantly associated with the presence of carotid plaque; HDL-C, LDL-C, Non-HDL-C levels, TC/HDL-C, LDL-C/HDL-C were significantly associated with the presence of common carotid artery (CCA) plaque. Compare with participants without carotid plaque, increased level of LDL-C/HDL-C was found in those with echolucent/polytype plaque. Similarly, compared with participants without CCA plaque, increased level of LDL-C/HDL-C was found in those with echolucent plaque. In conclusion, we found that serum HDL-C, Non-HDLc level, TC/HDLc, and LDLc/HDLc were all associated with the prevalence of carotid plaque, and LDL-C/HDL-C differed among different group of carotid plaque composition.

## Introduction

Cardiovascular disease (CVD), caused 17.7 million deaths in 2015, is the major cause of death worldwide^1^. Atherosclerosis is the leading contributor to CVD, and treatment of atherosclerosis is an essential step towards appropriate management and prevention of CVD^2^. Atherosclerosis is a systemic progression starting at young age and typically remains asymptomatic until late life, which could manifest in multiple vascular beds. Some researchers have demonstrated that atherosclerosis of peripheral vascular like carotid artery is one of the most significant prognostic predictors of cardiovascular morbidity and mortality^3,4^. Therefore, early detection and treatment of patients with carotid plaque may promote the prevention of CVD. Currently, the majority of carotid ultrasound studies have used carotid intima-medial thickness (IMT) as a surrogate marker for atherosclerosis. However, IMT is not as solid evidence for atherosclerotic infiltration in the arterial wall as significant plaque is^5^. Several studies indicated that the presence of plaque predicted the onset of cardiovascular and cerebrovascular diseases more accurately than the mean IMT, and the accuracy of this prediction was equivalent to that from pulse wave velocity in hypertensive patients^6–8^.

Lipid profiles have a pivotal role in the CVD pathophysiology and is an important modifiable risk factor for CVD^9^. Previous studies suggested that lipid profiles (i.e. TC, TG, HDL-C, LDL-C) were significantly associated with carotid plaque^10–14^. Other studies found that lipid profiles were associated with common carotid artery- intima-media thickness (CCA-IMT) thickening^15^, carotid intima-media thickness^13^, carotid femoral-pulse wave velocity (CF-PWV)^16^, or carotid intima-media roughness^17^. However, there have been few studies about the relationship between serum lipid profile and carotid plaque composition in Chinese Population. We therefore conducted the current study to investigate the association between various lipid profiles and the prevalence of carotid plaque and carotid plaque composition in a general Chinese population.

## Results

### Clinical data

A total of 3,214 participants (male: 2,212, females: 1,002) were included in the analysis. The characteristic of study participants are summarized by gender in Table 1. The mean age was 50.5 ± 10.8 years for men and 51.2 ± 10.8 years for women, respectively. Compared with women, men had generally higher glucose (mean: 5.38 vs. 5.16 mmol/L), TG (mean: 1.97 vs. 1.42 mmol/L), TC/HDL-C (mean: 3.91 vs. 3.38 mmol/L), LDL-C/HDL-C (mean: 2.47 vs. 2.17 mmol/L), TG/HDL-C (mean: 1.78 vs. 1.09 mmol/L), and CCA IMT (mean: 0.83 vs. 0.82 mm); lower HDL-C (mean: 1.30 vs. 1.50 mmol/L); and higher prevalence of carotid plaque (40.5% vs. 32.5%), CCA plaque (46.5% vs. 29.7%), ICA plaque (14.3% vs. 6.5%), and ECA plaque (3.7% vs. 1.4%). Additionally, the distribution of counts and the compositions of carotid artery plaques/CCA plaques were also different between genders (*P* < 0.001).

**Table 1:** General characteristic of study participants.

	Men		Women		*P*
	mean (SD)/ count (percentage)	N	mean (SD)/ count (percentage)	N	
Age, y	50.53(10.77)	2212	51.23(10.83)	1002	0.263
Height, cm	172.46(5.69)	1898	160.73(5.96)	821	0.372
Weight, kg	77.89(10.07)	1899	62.06(8.75)	823	<0.001
BMI, kg/m^2^	26.16(2.93)	1898	24.03(3.22)	821	0.001
SBP, mmHg	132.58(17.63)	1910	127.49(19.82)	823	<0.001
DBP, mmHg	82.26(12.86)	1909	74.61(12.83)	823	0.893
FPG, mmol/L	5.38(1.28)	2134	5.16(1.14)	951	<0.001
Uric acid, mmol/L	348.67(91.93)	2185	254.73(74.29)	985	<0.001
TG, mmol/L	1.97(1.75)	2212	1.42(0.96)	1002	<0.001
TC, mmol/L	4.67(1.08)	2192	4.71(1.06)	998	0.918
HDLc, mmol/L	1.30(0.47)	2209	1.50(0.47)	993	0.031
LDLc, mmol/L	2.95(0.87)	2164	2.99(0.81)	966	0.136
Non-HDLc, mmol/L	3.38(1.23)	2189	3.22(1.21)	989	0.594
TC/HDLc	3.91(1.28)	2189	3.38(1.04)	989	<0.001
LDLc/HDLc	2.47(0.87)	2161	2.17(0.74)	964	<0.001
TG/HDLc	1.78(2.02)	2209	1.09(1.01)	993	<0.001
CCA IMT, mm	0.83(0.08)	2212	0.82(0.06)	1002	<0.001
ICA IMT, mm	0.6(0.02)	2212	0.6(0.03)	1002	0.995
ECA IMT, mm	0.5(0.02)	2212	0.5(0.02)	1002	0.298
VA IMT, mm	0.2(0.05)	2212	0.2(0.02)	1002	0.918
Smoking	59(4.8%)	1231	0(0)	512	<0.001
Drinking	65(5.3%)	1231	1(0.2)	512	<0.001
Carotid plaque	10950(49.5%)	2212	326(32.5%)	1002	<0.001
CCA plaque	1028(46.5%)	2212	298(29.7%)	1002	<0.001
ICA plaque	317(14.3%)	2212	65(6.5%)	1002	<0.001
ECA plaque	81(3.7%)	2212	14(1.4%)	1002	<0.001
VA plaque	3(0.1%)	2212	2(0.2%)	1002	0.650
Carotid plaque composition*					<0.001
None	1117(50.5%)	2212	676(67.5)	1002	
Echolucent	435(19.7%)	2212	131(13.1)	1002	
Echogenic	15(0.7%)	2212	10(1)	1002	
Heterogeneous	297(13.4%)	2212	110(11.1)	1002	
Polytype	348(15.7%)	2212	75(7.5)	1002	
CCA plaque composition*					<0.001
None	1184(53.5%)	2212	704(70.3)	1002	
Echolucent	446(20.2%)	2212	127(12.7)	1002	
Echogenic	15(0.7%)	2212	8(0.8)	1002	
Heterogeneous	292(13.2%)	2212	101(10.1)	1002	
Polytype	275(12.4%)	2212	62(6.2)	1002	
No. of carotid plaque					<0.001
0	1117(50.5%)	2211	676(67.5)	1001	
1	436(19.7%)	2211	164(16.4)	1001	
2	275(12.4%)	2211	88(8.8)	1001	
≥3	383(17.3%)	2211	73(7.3)	1001	
No. of CCA plaque					<0.001
0	1184(53.5%)	2212	704(70.3)	1001	
1	464(21%)	2212	157(15.7)	1001	
2	305(13.8%)	2212	82(8.2)	1001	
≥3	259(11.7%)	2212	58(5.8)	1001	

BMI: body mass index; SBP: systolic blood pressure; FPG: fasting plasma glucose; DBP: diastolic blood pressure; TC: total cholesterol; TG: triglycerides; HDLc: high-density lipoprotein cholesterol; LDLc: low-density lipoprotein cholesterol; CCA: common carotid artery, ICA: internal carotid artery; ECA: external carotid artery; VA:Vertebral artery.

*plaque composition: if all the plaques had the same composition, subject was classified into with this plaque composition, if had different composition, subject was classified into with polytype plaques.

### Associations between lipid profiles and the odds of carotid plaque/CCA plaque

The associations between lipid profiles and the odds of carotid plaque/CCA plaque are presented in Table 2. For all participants, we detected statistically significant associations between: (1) higher HDL-C with lower odds of carotid plaque (fourth versus first quartile: OR, 0.63; 95% CI, 0.47–0.85; *P*-trend = 0.001) and CCA plaque (fourth versus first quartile: OR, 0.69; 95% CI, 0.52–0.93; *P*-trend = 0.009); (2) higher LDL-C with higher odds of CCA plaque (fourth versus first quartile: OR, 1.41; 95% CI, 1.09–1.84; *P*-trend = 0.001); (3) higher Non-HDL-C with higher odds of carotid plaque (fourth versus first quartile: OR, 1.36; 95% CI, 1.04–1.78; *P*-trend = 0.018) and CCA plaque (fourth versus first quartile: OR, 1.49; 95% CI, 1.13–1.97; *P*-trend = 0.002); (4) higher TC/HDL-C with higher odds of carotid plaque (fourth versus first quartile: OR, 1.47; 95% CI, 1.12–1.94; *P*-trend = 0.004) and CCA plaque (fourth versus first quartile: OR, 1.49; 95% CI, 1.13–1.97; *P*-trend = 0.002); (5) higher LDL-C/HDL-C with higher odds of carotid plaque (fourth versus first quartile: OR, 1.74; 95% CI, 1.32–2.29; *P*-trend < 0.001) and CCA plaque (fourth versus first quartile: OR, 1.72; 95% CI, 1.31–2.26; *P*-trend < 0.001).

**Table 2:** Association of plasma lipid profiles and odds of carotid plaque.

	Odds ratios (95% CI) for carotid plaque					Odds ratios (95% CI) for common carotid artery plaque				
	Q1	Q2	Q3	Q4	P-trend	Q1	Q2	Q3	Q4	p-trend
TG										
Cases/N	684	678	677	680		680	670	686	662	
Model 1	1.00	1.05(0.81–1.35)	1.05(0.81–1.35)	1.19(0.92–1.53)	0.199	1.00	1.07(0.83–1.38)	1.07(0.82–1.38)	1.22 (0.95–1.58)	0.135
Model 2	1.00	0.87(0.65–1.14)	0.82(0.61–1.10)	0.85(0.63–1.15)	0.309	1.00	0.90(0.68–1.20)	0.84(0.63–1.13)	0.90 (0.66–1.22)	0.471
TC										
Cases/N	680	670	686	662		680	670	686	662	
Model 1	1.00	0.96(0.75–1.24)	1.16(0.91–1.49)	1.25 (0.98–1.60)	0.029	1.00	0.98(0.76–1.27)	1.28(0.99–1.64)	1.35(1.05–1.74)*	0.004
Model 2	1.00	0.94(0.72–1.23)	1.10(0.85–1.43)	1.16(0.89–1.52)	0.140	1.00	0.96(0.73–1.25)	1.22(0.93–1.59)	1.25(0.96–1.64)	0.030
HDLc										
Cases/N	685	658	707	659		685	658	707	659	
Model 1	1.00	1.01(0.79–1.29)	0.83(0.65–1.06)	0.74(0.58–0.95)*	0.008	1.00	0.96(0.75–1.23)	0.89(0.70–1.14)	0.77(0.60–0.99)*	0.040
Model 2	1.00	0.93(0.72–1.21)	0.74(0.57–0.97)*	0.63(0.47–0.85)**	0.001	1.00	0.92(0.71–1.20)	0.81(0.62–1.07)	0.69(0.52–0.93)*	0.009
LDLc										
Cases/N	679	656	671	649		679	656	671	649	
Model 1	1.00	0.81(0.63–1.05)	1.22(0.95–1.56)	1.35(1.05–1.73)*	0.001	1.00	0.82(0.63–1.06)	1.23(0.96–1.58)	1.44(1.12–1.85)**	<0.001
Model 2	1.00	0.77(0.59–1.01)	1.12(0.87–1.46)	1.30(0.99–1.69)	0.005	1.00	0.79(0.60–1.03)	1.15 (0.89–1.50)	1.41(1.09–1.84)*	0.001
Non-HDLc										
Cases/N	670	681	678	659		670	681	678	659	
Model 1	1.00	1.06(0.82–1.37)	1.27(0.99–1.64)	1.45(1.12–1.86)**	0.001	1.00	1.13(0.87–1.46)	1.44(1.11–1.85)**	1.59(1.23–2.05)***	<0.001
Model 2	1.00	1.06(0.81–1.39)	1.18(0.90–1.54)	1.36(1.04–1.78)*	0.018	1.00	1.13(0.86–1.49)	1.33(1.02–1.75)*	1.49(1.13–1.97)**	0.002
TC/HDLc										
Cases/N	677	668	676	667		677	668	676	667	
Model 1	1.00	1.08(0.84–1.39)	1.25(0.97–1.62)	1.50(1.16–1.93)**	0.001	1.00	1.05(0.81–1.35)	1.31(1.01–1.69)*	1.53(1.19–1.98)**	<0.001
Model 2	1.00	1.10(0.84–1.44)	1.19(0.91–1.57)	1.47(1.12–1.94)**	0.004	1.00	1.06(0.80–1.39)	1.24(0.94–1.63)	1.49(1.13–1.97)**	0.002
LDLc/HDLc										
Cases/N	675	676	673	685		676	664	659	652	
Model 1	1.00	1.28(0.99–1.65)	1.16(0.90–1.50)	1.76(1.36–2.27)***	<0.001	1.00	1.22(0.95–1.58)	1.21(0.93–1.56)	1.76(1.36–2.27)***	<0.001
Model 2	1.00	1.22(0.93–1.59)	1.10(0.84–1.45)	1.74(1.32–2.29)***	<0.001	1.00	1.17(0.89–1.53)	1.14(0.87–1.50)	1.72(1.31–2.26)***	<0.001
TG/HDLc										
Cases/N	684	678	677	680		675	676	673	685	
Model 1	1.00	1.06(0.82–1.37)	1.14(0.89–1.47)	1.24(0.96–1.60)	0.077	1.00	1.02(0.79–1.32)	1.11(0.86–1.44)	1.22(0.95–1.58)	0.092
Model 2	1.00	0.98(0.75–1.29)	1.05 (0.79–1.38)	1.08(0.81–1.43)	0.524	1.00	0.94(0.71–1.24)	1.01(0.76–1.34)	1.04(0.79–1.39)	0.625

TC: total cholesterol; TG: triglycerides; HDLc: high-density lipoprotein cholesterol; LDLc: low-density lipoprotein cholesterol; CCA: common carotid artery, ICA: internal carotid artery; ECA: external carotid artery; Q1: Quantiles 1; Q2: Quantiles 2; Q3: Quantiles 3; Q4: Quantiles 4.

Model 1: adjusted for age (years, continuous variable), sex (male or female), and BMI (continuous variable); Model 2: further adjusted for systolic blood pressure (SBP), diastolic blood pressure (DBP), fasting plasma glucose, uric acid, TC (not for TC vs. atherosclerosis), TG (not for TG vs. atherosclerosis), HDLc(not for HDLc vs. atherosclerosis) and LDLc (not for LDLc vs. atherosclerosis)

Compared with the lowest quarter: *P < 0.05, **P ≤ 0.01, ***P ≤ 0.001.

### Association between lipid profiles and carotid plaque composition

The covariate-adjusted mean lipid profiles according to composition of carotid artery/CCA plaques were presented in Tables 3 and 4. Compare with participants without carotid plaque, increased level of LDL-C/HDL-C was found in those with echolucent/polytype plaque. Similarly, compared with participants without CCA plaque, increased level of LDL-C/HDL-C was found in those with echolucent plaque.

**Table 3:** Covariate-adjusted mean (SEM) lipid profiles according to different composition of carotid plaque.

	Carotid plaque composition																
	n	None	SE	n	Echolucent	SE	n	Echogenic	SE	n	Heterogeneous	SE	n	Polytype	SE	*P*	*P*-trend
		mean			mean			mean			mean			mean			
TG																	
Model 1	1,512	1.8	0.04	485	1.84	0.07	20	1.54	0.34	334	1.82	0.09	368	1.79	0.09	0.920	0.832
Model 2	1,360	1.86	0.04	427	1.73	0.07	19	1.58	0.31	299	1.76	0.08	321	1.78	0.08	0.452	0.56
TC																	
Model 1	1,502	4.64	0.03	479	4.74	0.05	20	4.36	0.24	331	4.71	0.06	366	4.75	0.06	0.183	0.246
Model 2	1,386	4.66	0.03	435	4.73	0.05	19	4.32	0.23	303	4.7	0.06	324	4.74	0.06	0.318	0.431
HDLc																	
Model 1	1,504	1.37	0.01	484	1.35	0.02	20	1.3	0.1	333	1.36	0.03	368	1.33	0.03	0.711	0.463
Model 2	1,360	1.33	0.01	427	1.284	0.02	19	1.3	0.08	299	1.29	0.02	321	1.29	0.02	0.089	0.179
LDLc																	
Model 1	1,474	2.9	0.02	**473**	3.05*	**0.04**	20	2.68	0.19	327	2.97	0.05	361	3.05	0.05	**0.002**	0.08
Model 2	1,360	2.9	0.02	**427**	3.05*	**0.04**	19	2.62	0.19	299	2.97	0.05	321	3.05	0.05	**0.004**	0.116
Non-HDLc																	
Model 1	1,494	3.28	0.03	478	3.41	0.05	20	3.06	0.26	330	3.36	0.07	366	3.42	0.07	0.132	0.172
Model 2	1,380	3.3	0.03	435	3.4	0.05	19	3.01	0.25	303	3.37	0.07	324	3.42	0.06	0.245	0.259
TC/HDLc																	
Model 1	1,494	3.68	0.03	478	3.82	0.05	20	3.49	0.26	330	3.79	0.07	**366**	3.9*	**0.06**	**0.016**	**0.02**
Model 2	1,380	3.7	0.03	435	3.82	0.05	19	3.45	0.25	303	3.8	0.07	324	3.9	0.06	**0.042**	**0.031**
LDLc/HDLc																	
Model 1	1,472	2.31	0.02	**472**	2.46**	**0.04**	20	2.13	0.18	326	2.41	0.05	**361**	2.51**	**0.05**	<**0.001**	**0.005**
Model 2	1,360	2.31	0.02	**427**	2.47**	**0.04**	19	2.09	0.18	299	2.43	0.05	**321**	2.51**	**0.05**	<**0.001**	**0.007**
TG/HDLc																	
Model 1	1,504	1.59	0.05	484	1.56	0.08	20	1.44	0.39	333	1.55	0.1	368	1.61	0.1	0.975	0.871
Model 2	1,388	1.58	0.02	437	1.59	0.03	19	1.73	0.13	304	1.59	0.03	325	1.63	0.03	0.592	0.291

Compared with participants without CCA plaque, *P < 0.05, **P ≤ 0.01, ***P ≤ 0.001.

**Table 4:** Covariate-adjusted mean (SEM) lipid profiles according to different composition of common carotid artery plaque.

	n	None	SE	n	Echolucent	SE	n	Echogenic	SE	n	Heterogeneous	SE	n	Polytype	SE	*P*	*P*-trend
		mean			mean			mean			mean			mean			
TG																	
Model 1	1590	1.81	0.04	492	1.77	0.07	16	1.44	0.39	328	1.83	0.09	293	1.88	0.09	0.784	0.438
Model 2	1432	1.84	0.04	431	1.72	0.07	15	1.57	0.35	291	1.77	0.08	257	1.83	0.09	0.527	0.908
TC																	
Model 1	1579	4.67	1.04	487	4.72	1.09	16	4.19	0.91	325	4.65	1.01	291	4.70	1.14	0.090	0.103
Model 2	1458	4.66	0.03	440	4.73	0.05	15	4.28	0.26	295	4.72	0.06	259	4.72	0.07	0.336	0.525
HDLc																	
Model 1	1582	1.36	0.01	491	1.35	0.02	16	1.34	0.12	327	1.36	0.03	293	1.33	0.03	0.792	0.415
Model 2	1432	1.33	0.01	431	1.29	0.02	15	1.32	0.09	291	1.29	0.02	257	1.29	0.02	0.269	0.214
LDLc																	
Model 1	1551	2.90	0.02	**479**	3.05**	**0.04**	16	2.58	0.21	321	2.98	0.05	288	3.05	0.05	**0.001**	0.087
Model 2	1432	2.90	0.02	**431**	3.06*	**0.04**	15	2.56	0.21	291	3.00	0.05	257	3.02	0.05	**0.003**	0.232
Non-HDLc																	
Model 1	1571	3.28	0.03	486	3.39	0.05	16	2.91	0.30	324	3.38	0.07	291	3.46	0.07	0.078	0.067
Model 2	1452	3.31	0.03	440	3.39	0.05	15	2.96	0.28	295	3.39	0.07	259	3.40	0.07	0.330	0.346
TC/HDLc																	
Model 1	1571	3.68	0.03	486	3.78	0.05	16	3.32	0.29	324	3.81	0.07	**291**	3.96**	0.07	**0.003**	**0.001**
Model 2	1452	3.71	0.03	440	3.79	0.05	15	3.38	0.28	295	3.84	0.07	259	3.91	0.07	0.053	**0.017**
LDLc/HDLc																	
Model 1	1549	2.31	0.02	**478**	2.45**	**0.04**	16	2.00	0.20	320	2.43	0.05	**288**	2.53**	**0.05**	<**0.001**	**0.001**
Model 2	1432	2.32	0.02	**431**	2.47**	**0.04**	15	2.05	0.20	291	2.46	0.05	**257**	2.49*	**0.05**	**<0.001**	**0.012**
TG/HDLc																	
Model 1	1582	1.58	0.05	491	1.52	0.08	16	1.31	0.44	327	1.57	0.10	293	1.71	0.11	0.638	0.276
Model 2	1432	1.62	0.05	431	1.55	0.08	15	1.33	0.45	291	1.60	0.11	257	1.72	0.11	0.739	0.395

Compared with participants without CCA plaque, *P < 0.05, **P ≤ 0.01, ***P ≤ 0.001.

### Association between lipid profiles and carotid plaque number

The covariate-adjusted mean lipid profiles according to number of carotid artery/CCA plaques were presented in Tables S1, S2. Compare with participants without carotid plaque, increased level of LDLc/HDLc was found in those with ≥3 carotid plaque. Similarly, compared with participants without CCA plaque, increased level of LDL-C/HDL-C was found in those with ≥3 CCA plaque.

### Subgroup analyses

The subgroup analyses generally yielded results consistent with the above findings (Table S3). There was no significant effect modification by sex or BMI on the association between lipid profiles and carotid plaque/CCA plaque (all *P*-values were greater than 0.10).

## Discussion

To the best of our knowledge, this is the largest study in China to investigate the association between lipid profiles and the prevalence of carotid plaque and carotid plaque composition. The main results are as follows: serum HDL-C level was negatively associated with odds of carotid plaque and CCA plaque; serum LDL-C level was positively associated with odds of CCA plaque; serum Non-HDLc level, TC/HDLc, and LDLc/HDLc were positively associated with odds of both carotid plaque and CCA plaque. LDL-C/HDL-C differed among different group of carotid plaque composition.

Advanced atherosclerotic plaques are characterized as lipid nucleus which were covered with fibrous caps consisting of extracellular matrix and smooth muscle cells^18^. Several procedure associated with the progression of the atherosclerotic lesions(e.g. lipids accumulation, thrombosis, proteolysis, inflammation, and apoptosis) might be relevant with the development of plaque. It has been demonstrated that carotid plaque, especially the thick and irregular ones were risks factor for cardiovascular disease^19–21^. Therefore, identification of serum biomarkers related to the presence of plaque would be of great advantage in the prevention of atherosclerosis and CVD. The various lipid profiles examined in our study were related with carotid plaque, thus might be correlated with CVD. Previous studies suggested that lipid profiles have pivotal roles in the pathophysiology of carotid plaque^10–17^, which is consistent with our study. In contrast, one study in USA found no significant association between HDL-C or TG and carotid plaque, but the authors reported that higher TC, LDL, or non-HDL was associated with higher risk of carotid plaque^22^. An Algeria cross-sectional study also suggested inconsistent findings: lower HDL-C is associated with higher prevalence level of carotid plaque, but not LDL-C^23^. The inconsistency might be ascribed to many factors, including the study population heterogeneity, various study regions and different study designs.

Ultrasound (US), a non-radiative and cost-effective technique, is a patient-friendly an ideal method for immediate diagnosis and follow-up^24^. Previous studies have demonstrated that hypoechoic plaques (echolucent) are more often associated with cardiovascular symptoms than hyperechoic ones (echorich). The meta-analysis conducted by Jashari *et al*. demonstrated that carotid plaque echogenicity was associated with increased risk of CV events^25^. However, evidence regarding the relationship between serum lipid profile and carotid plaque composition in Chinese Population is limited. Our study found that LDL-C/HDL-C level was increased in those with echolucent plaque than those without carotid plaque. The analysis from our study provides new evidence for the associations between plaque characterization/features and health related outcomes.

The potential pathophysiological mechanisms through which lipid profiles might increase the risk of carotid plaque are not fully understood. First, the main beneficial effect of HDL cholesterol is to reverse the transport of cholesterol, remove free cholesterol from endothelial macrophages, and return it to the liver for excretion into bile, thus preventing the formation of atherosclerotic plaques^26^. Second, it has been demonstrated that HDL-C has both direct and indirect anti-inflammatory effects with potential antithrombotic results^27^.Third, the LDL-C/HDL-C ratio is closely correlated with the distribution of HDL-C subclasses. With the increase of LDL-C/HDL-C ratio, the size of HDL-C particles generally decreases, suggesting that the maturation process of high density lipoprotein is blocked, which may be one of the contributors for the progress of atherosclerosis^28^.

There were some strengths in this study. First, we collected information on several potential confounders, and the potential effects of them were taken into account. Second, we firstly investigated the association of various lipid parameters and carotid plaque composition in Chinese Population. Third, subgroup analyses generally yielded similar results, indicating that the associations are robust.

Despite these strengths, the study also had several limitations. First, this is a cross-sectional analysis, thus it is difficult to detect the temporal relationship. Second, our study was carried out in a single-center, and these finding should be verified in multi-center study with larger populations. Last, we used ultrasound to assess the presence of plaque, which may be less reliable than the high-resolution magnetic resonance imaging (MRI) or computed tomography (CT). However, one study investigating the accuracy of ultrasound, CT, and MRI in diagnosis of carotid plaque morphology found that ultrasound has higher accuracy for diagnostics of carotid plaque morphology than CT or MRI^29^. In addition, compared with CT or MRI, ultrasound is safe, noninvasive, and inexpensive^30^.

In summary, our study demonstrated that serum HDL-C level was associated with lower odds of carotid plaque and CCA plaque; serum LDL-C level was associated with higher odds of CCA plaque; serum non-HDL-C level, TC/HDL-C ratio, and LDL-C/HDL-C ratio were associated with higher odds of carotid plaque and CCA plaque. LDL-C/HDL-C differed among different group of carotid plaque composition. Further prospective studies with larger sample size are needed to verify these associations.

## Methods

### Study population

During January 2015 and December 2017, 3,214 consecutive participants (2,212 men, 1,002 women) who underwent a standard medical screening at the Physical Center of the First Affiliated Hospital of Zhengzhou University, China, were included. We excluded participants with the following conditions that might seriously influence carotid plaque and lipid profiles: (1) aged less than 18 years old; (2) with a history of cardiovascular disease, peripheral arterial disease, oncology disease, infectious disease, or serious liver or renal disease; (3) without data on lipid profiles or carotid ultrasound measurements; (4) taking medicine when undergoing the medical examination.

### Lipid profiles assessment

Overnight fast blood samples were collected in the morning. Plasma levels of TC, triglycerides (TG), HDL-C, and LDL-C were measured by a fully automatic analyzer, ROCHE (Roche Molecular Systems, Inc. Basel, Switzerland) module Cobas 8000 (C701/C702/C502), and kits were procured by ROCHE. Quality control is maintained by the laboratory with standard procedures. The coefficient of variation for repeated measurements on samples from individual hospitalized patients is maintained at ≤2.5%.

### Carotid ultrasound assessment

Carotid ultrasound was assessed by B-mode ultrasound with an Aplio 300 ultrasound system (Toshiba Medical Systems, Tokyo, Japan) by trained and certified technologists. With the subject lying in a supine position, the extracranial carotid arteries were imaged in the longitudinal (anterior, lateral, and posterior views) and transverse planes, the common carotid artery (CCA), the internal carotid artery (ICA), and the external carotid artery (ECA) on both sides were carefully scanned under B-mode imaging from multiple directions. The IMT was defined as the distance between the leading edge of the lumen-intima echo and the leading edge of the media adventitia echo. The carotid intima-media thickness (CIMT) was defined as the mean of the right and left IMT of the CCA.

Carotid plaque is defined as the presence of focal wall thickening that is at least 0.5 mm, or 50% greater than that of the surrounding vessel wall, or as a focal region with CIMT greater than 1.5 mm that protrudes into the lumen that is distinct from the adjacent boundary^31^. Plaques were classified as echolucent (low grayscale median, lipid rich), echogenic (high grayscale median, mostly occupied by calcified areas), and heterogeneous (mixed echolucent and echogenic)^32^. If all the plaques had the same composition, the participant was classified as plaque composition, those who had different composition were defined as polytype plaques. All carotid artery plaque measurement was conducted by the same trained physician using the same B-mode ultrasound. The study adopted strict quality control procedures for monitoring and testing consistency in image acquisition and image analysis.

### Covariates

A standard physician-administered questionnaire was used to collected information on the demographic characteristics, lifestyle factors, and medical history by trained physicians. Participants who smoked at least one cigarette per day or drank alcohol once a week for at least six months were defined as smokers or drinkers. Weight and height were measured on an auto-anthropometer with subjects wearing light clothing and no shoes. Body mass index (BMI) was calculated as the weight (kg) divided by the square of the height (m). Blood pressure was measured using an electronic device by trained physicians after the subjects sitting relaxed for 10 mins.

### Statistical analyses

Descriptive data are presented as mean (standard deviation) for continuous variables and number (percentage) for categorical variables.

Logistic regression model was used to examine the association between plasma lipid profiles and odds of carotid plaque. And two models were developed: Model 1: adjusted for age (years, continuous variable), sex (male or female), and BMI (continuous variable, kg/m^2^); Model 2: further adjusted for systolic blood pressure (SBP), diastolic blood pressure (DBP), fasting plasma glucose, uric acid, TC (not for TC vs. carotid plaque), TG (not for TG vs. carotid plaque), HDL-C (not for HDL-C vs. carotid plaque) and LDL-C (not for LDL-C vs. carotid plaque).

Analysis of covariance (ANCOVA) was used to compare the mean plasma lipid profiles among different number and composition of carotid artery plaques. The two aforementioned models were also adopted. Subgroup analyses were conducted to investigate whether the relationships between plasma lipid profiles and carotid plaque were modified by sex (men or women) or BMI (<25 kg/m^2^ or ≥25 kg/m^2^). Each potential modifier was examined in a separate model by adding a multiplicative interaction term (i.e. potential modifier * lipid profiles).

All analyses were performed with SPSS 21.0 for Windows (SPSS, Inc., Chicago, USA). Two-sided bonferroni correction *P* values < 0.01 for the difference of covariate-adjusted mean (SEM) among different composition/number of carotid plaque (i.e. None, Echolucent, Echogenic, Heterogeneous, and Polytype). Two-side *P* values < 0.05 were considered statistically significant for the other associations.

### Ethical approval

Ethical approvals for the study were obtained from the ethics committees of the First Affiliated Hospital of Zhengzhou University. Informed consent was obtained from all participants before they participate the study. And all procedures were performed in accordance with the approved guidelines laid down in the Declaration of Helsinki.

## Supplementary information

Supplemental Tables

## Data Availability

Additional data are available from the corresponding author for reasonable requesting.
