# Supplementary material for: Association between lipid profiles and presence of carotid plaque
Source: Sci Rep. 2019 Nov 29;9:18011. doi: 10.1038/s41598-019-54285-w (PMC6884522; doi:10.1038/s41598-019-54285-w)
Supplement: Supplementary file 1 — Supplemental Tables [file 41598_2019_54285_MOESM1_ESM.docx]

# Association between lipid profiles and presence of carotid plaque

Yanhua Liu^a#^, Yongjian Zhu^b#^, Wenrui Jia^C^, Dan Sun^C^, Li Zhao^C^, Chen Zhang^C^, Cuicui Wang^C^, Gaiyun Chen^a^, Sanxian Fu^a^, Yacong Bo^a^, Yurong Xing^C*^

^a^Department of Nutrition, the First Affiliated Hospital of Zhengzhou University, Zhengzhou, 450052.

^b^Department of Cardiology, the First Affiliated Hospital of Zhengzhou University, Zhengzhou, 450052.

^c^Depatment of Physical Center, the First Affiliated Hospital of Zhengzhou University, Zhengzhou, 450052.

#Yanhua Liu and Yongjian Zhu contributed equally to this work.

*Corresponding author: Professor Yurong Xing, Department of Physical Center, the First Affiliated Hospital of Zhengzhou University, No. 1 Jianshe East Road, Zhengzhou, 450000, Tel: +86-15737150025; Fax: 86-37166862963, E-mail addresses: [405274775@qq.com](mailto:405274775@qq.com).

Table S1. Covariate-adjusted mean (SEM) lipid profiles according to number of carotid plaque

|  | Number of carotid plaque composition | | | | | | | | | | | | | |
| --- | --- | --- | --- | --- | --- | --- | --- | --- | --- | --- | --- | --- | --- | --- |
|  | None | | | 1 | | | 2 | | | ≥3 | | | *P* | *P*-trend |
|  | n | mean | SE | n | mean | SE | n | mean | SE | n | mean | SE |  |  |
| TG |  |  |  |  |  |  |  |  |  |  |  |  |  |  |
| Model 1 | 1512 | 1.80 | 0.04 | 516 | 1.87 | 0.07 | 299 | 1.75 | 0.09 | 391 | 1.80 | 0.09 | 0.760 | 0.670 |
| Model 2 | 1360 | 1.86 | 0.04 | 461 | 1.73 | 0.06 | 258 | 1.76 | 0.09 | 347 | 1.75 | 0.08 | 0.360 | 0.359 |
| TC |  |  |  |  |  |  |  |  |  |  |  |  |  |  |
| Model 1 | 1502 | 4.64 | 0.03 | 507 | 4.69 | 0.05 | 299 | 4.73 | 0.06 | 389 | 4.79 | 0.06 | 0.154 | **0.028** |
| Model 2 | 1386 | 4.66 | 0.03 | 467 | 4.69 | 0.05 | 263 | 4.76 | 0.06 | 351 | 4.73 | 0.06 | 0.493 | 0.218 |
| HDLc |  |  |  |  |  |  |  |  |  |  |  |  |  |  |
| Model 1 | 1504 | 1.37 | 0.01 | 514 | 1.35 | 0.02 | 299 | 1.36 | 0.03 | 391 | 1.32 | 0.03 | 0.428 | 0.181 |
| Model 2 | 1360 | 1.33 | 0.01 | 461 | 1.29 | 0.02 | 258 | 1.29 | 0.02 | 347 | 1.28 | 0.02 | 0.047 | 0.053 |
| LDLc |  |  |  |  |  |  |  |  |  |  |  |  |  |  |
| Model 1 | 1474 | 2.89 | 0.02 | 506 | 2.97 | 0.04 | **290** | **3.05*** | **0.050** | **384** | **3.08**** | **0.047** | **0.002** | **<0.001** |
| Model 2 | 1360 | 2.90 | 0.02 | 461 | 2.98 | 0.04 | **258** | **3.06*** | **0.053** | **347** | **3.06*** | **0.049** | **0.009** | **0.003** |
| Non-HDLc |  |  |  |  |  |  |  |  |  |  |  |  |  |  |
| Model 1 | 1494 | 3.27 | 0.03 | 505 | 3.36 | 0.05 | 299 | 3.37 | 0.07 | 389 | 3.47 | 0.07 | 0.073 | **0.019** |
| Model 2 | 1380 | 3.30 | 0.03 | 467 | 3.36 | 0.05 | 263 | 3.42 | 0.07 | 351 | 3.40 | 0.06 | 0.340 | 0.148 |
| TC/HDLc |  |  |  |  |  |  |  |  |  |  |  |  |  |  |
| Model 1 | 1494 | 3.67 | 0.03 | 505 | 3.79 | 0.05 | 299 | 3.79 | 0.07 | **389** | **3.93**** | **0.064** | **0.006** | **0.002** |
| Model 2 | 1380 | 3.70 | 0.03 | 467 | 3.79 | 0.05 | 263 | 3.84 | 0.07 | 351 | 3.88 | 0.06 | 0.056 | **0.013** |
| LDLc/HDLc |  |  |  |  |  |  |  |  |  |  |  |  |  |  |
| Model 1 | 1472 | 2.30 | 0.02 | 504 | 2.40 | 0.04 | **290** | **2.46*** | **0.048** | **384** | **2.54***** | **0.045** | **<0.001** | **<0.001** |
| Model 2 | 1360 | 2.31 | 0.02 | 461 | 2.42 | 0.04 | **258** | **2.48*** | **0.020** | **347** | **2.53***** | **0.046** | **<0.001** | **<0.001** |
| TG/HDLc |  |  |  |  |  |  |  |  |  |  |  |  |  |  |
| Model 1 | 1504 | 1.59 | 0.05 | 514 | 1.56 | 0.08 | 299 | 1.54 | 0.10 | 391 | 1.61 | 0.10 | 0.949 | 0.887 |
| Model 2 | 1360 | 1.63 | 0.05 | 461 | 1.56 | 0.08 | 258 | 1.62 | 0.11 | 347 | 1.62 | 0.10 | 0.908 | 0.943 |

Compared with participants without CCA plaque, **P*<0.05,***P*≤0.01,*** *P*≤0.001

Table S2. Covariate-adjusted mean (SEM) lipid profiles according to number of common carotid artery plaque

|  | Number of common carotid plaque composition | | | | | | | | | | | | | |
| --- | --- | --- | --- | --- | --- | --- | --- | --- | --- | --- | --- | --- | --- | --- |
|  | None | | | 1 | | | 2 | | | ≥3 | | | *P* | *P*-trend |
|  | n | mean | SE | n | mean | SE | n | mean | SE | n | mean | SE |  |  |
| TG |  |  |  |  |  |  |  |  |  |  |  |  |  |  |
| Model 1 | 1590 | 1.81 | 0.04 | 532 | 1.84 | 0.07 | 323 | 1.79 | 0.09 | 274 | 1.75 | 0.10 | 0.885 | 0.508 |
| Model 2 | 1432 | 1.84 | 0.04 | 469 | 1.75 | 0.06 | 285 | 1.80 | 0.08 | 240 | 1.74 | 0.09 | 0.537 | 0.409 |
| TC |  |  |  |  |  |  |  |  |  |  |  |  |  |  |
| Model 1 | 1579 | 4.64 | 0.03 | 524 | 4.70 | 0.05 | 323 | 4.72 | 0.06 | 272 | 4.83 | 0.07 | 0.070 | **0.012** |
| Model 2 | 1458 | 4.66 | 0.03 | 476 | 4.71 | 0.05 | 291 | 4.72 | 0.06 | 242 | 4.76 | 0.07 | 0.556 | 0.208 |
| HDLc |  |  |  |  |  |  |  |  |  |  |  |  |  |  |
| Model 1 | 1582 | 1.37 | 0.01 | 530 | 1.36 | 0.02 | 323 | 1.35 | 0.03 | 274 | 1.33 | 0.03 | 0.764 | 0.300 |
| Model 2 | 1432 | 1.33 | 0.01 | 469 | 1.30 | 0.02 | 285 | 1.29 | 0.02 | 240 | 1.29 | 0.02 | 0.163 | 0.136 |
| LDLc |  |  |  |  |  |  |  |  |  |  |  |  |  |  |
| Model 1 | 1551 | 2.90 | 0.02 | 519 | 2.99 | 0.04 | 315 | 3.02 | 0.05 | **270** | **3.12**** | **0.054** | **0.001** | **<0.001** |
| Model 2 | 1432 | 2.90 | 0.02 | 469 | 3.01 | 0.04 | 285 | 3.02 | 0.05 | **240** | **3.08*** | **0.057** | **0.009** | **0.006** |
| Non-HDLc |  |  |  |  |  |  |  |  |  |  |  |  |  |  |
| Model 1 | 1571 | 3.28 | 0.03 | 522 | 3.36 | 0.05 | 323 | 3.37 | 0.07 | **272** | **3.51*** | **0.076** | 0.054 | **0.012** |
| Model 2 | 1452 | 3.31 | 0.03 | 476 | 3.37 | 0.05 | 291 | 3.38 | 0.07 | 242 | 3.42 | 0.07 | 0.465 | 0.198 |
| TC/HDLc |  |  |  |  |  |  |  |  |  |  |  |  |  |  |
| Model 1 | 1571 | 3.68 | 0.03 | 522 | 3.78 | 0.05 | 323 | 3.82 | 0.07 | **272** | **3.95**** | **0.074** | **0.009** | **0.002** |
| Model 2 | 1452 | 3.71 | 0.03 | 476 | 3.79 | 0.05 | 291 | 3.84 | 0.07 | 242 | 3.89 | 0.07 | 0.107 | **0.031** |
| LDLc/HDLc |  |  |  |  |  |  |  |  |  |  |  |  |  |  |
| Model 1 | 1549 | 2.31 | 0.02 | 517 | 2.42 | 0.04 | **315** | **2.46*** | **0.047** | **270** | **2.56***** | **0.052** | **<0.001** | **<0.001** |
| Model 2 | 1432 | 2.32 | 0.02 | **469** | **2.43** | **0.036*** | **285** | **2.47*** | **0.048** | **240** | **2.54**** | **0.053** | **<0.001** | **<0.001** |
| TG/HDLc |  |  |  |  |  |  |  |  |  |  |  |  |  |  |
| Model 1 | 1582 | 1.58 | 0.05 | 530 | 1.57 | 0.08 | 323 | 1.60 | 0.10 | 274 | 1.55 | 0.11 | 0.987 | 0.872 |
| Model 2 | 1432 | 1.62 | 0.05 | 469 | 1.58 | 0.08 | 285 | 1.66 | 0.11 | 240 | 1.58 | 0.12 | 0.906 | 0.932 |

Compared with participants without CCA plaque, **P*<0.05,***P*≤0.01,*** *P*≤0.001

Table S3. Stratified analysis of association between plasma lipid profiles and prevalence of carotid plaque

|  | Odds ratios (95% CI) for carotid plaque | | | | |  | Odds ratios (95% CI) for common carotid artery plaque | | | | |
| --- | --- | --- | --- | --- | --- | --- | --- | --- | --- | --- | --- |
|  | Q1 | Q2 | Q3 | Q4 | *P* |  | Q1 | Q2 | Q3 | Q4 | *P* |
| TG |  |  |  |  |  |  |  |  |  |  |  |
| Sex |  |  |  |  | *0.714^$^* |  |  |  |  |  | *0.258^$^* |
| Male | 1.00 | 0.89(0.65-1.22) | 0.92(0.66-1.28) | 0.87(0.61-1.23) | 0.501 |  | 1.00 | 0.91(0.66-1.26) | 0.96(0.69-1.34) | 0.96(0.68-1.35) | 0.907 |
| Female | 1.00 | 0.75(0.42-1.34) | 0.52(0.28-0.96)* | 0.67(0.34-1.26) | 0.180 |  | 1.00 | 0.81 (0.45-1.48) | 0.48(0.26-0.91)* | 0.62(0.32-1.19) | 0.088 |
| BMI |  |  |  |  | *0.699^$^* |  |  |  |  |  | *0.180^$^* |
| <25 | 1.00 | 0.75(0.49-1.14) | 0.87(0.55-1.37) | 0.77(0.47-1.26) | 0.426 |  | 1.00 | 0.81(0.53-1.24) | 0.85(0.53-1.34) | 0.70(0.42-1.16) | 0.220 |
| ≥25 | 1.00 | 0.934(0.64-1.38) | 0.78(0.53-1.14) | 0.87(0.59-1.29) | 0.422 |  | 1.00 | 0.97(0.66-1.42) | 0.83(0.57-1.23) | 0.99(0.67-1.48) | 0.928 |
| TC |  |  |  |  |  |  |  |  |  |  |  |
| Sex |  |  |  |  | *0.136^$^* |  |  |  |  |  | *0.180^$^* |
| Male | 1.00 | 0.88(0.65-1.20) | 1.00(0.73-1.35) | 1.07(0.78-1.46) | 0.498 |  | 1.00 | 0.91(0.67-1.24) | 1.15(0.85-1.56) | 1.15(0.84-1.57) | 0.184 |
| Female | 1.00 | 1.15(0.66-2.01) | 1.55(0.91-2.65) | 1.51(0.90-2.56) | 0.073 |  | 1.00 | 1.15(0.64-2.08) | 1.54(0.88-2.69) | 1.67(0.97-2.88) | 0.039 |
| BMI |  |  |  |  | *0.810^$^* |  |  |  |  |  | *0.609^$^* |
| <25 | 1.00 | 0.97(0.62-1.51) | 1.21(0.79-1.84) | 1.19(0.77-1.82) | 0.282 |  | 1.00 | 1.09(0.69-1.71) | 1.31(0.84-2.02) | 1.38(0.89-2.14) | 0.104 |
| ≥25 | 1.00 | 0.91(0.65-1.28) | 1.06(0.75-1.48) | 1.15(0.82-1.63) | 0.278 |  | 1.00 | 0.88(0.63-1.24) | 1.19(0.84-1.66) | 1.19(0.84-1.68) | 0.129 |
| HDLc |  |  |  |  |  |  |  |  |  |  |  |
| Sex |  |  |  |  | *0.454^$^* |  |  |  |  |  | *0.154^$^* |
| Male | 1.00 | 0.99 (0.73-1.35) | 0.69(0.51-0.95)* | 0.65 (0.47-0.91)* | 0.002 |  | 1.00 | 0.93(0.69-1.27) | 0.71(0.52-0.97)* | 0.67(0.48-0.94)* | 0.006 |
| Female | 1.00 | 0.74(0.44-1.24) | 0.88(0.51-1.51) | 0.55(0.30-0.99)* | 0.106 |  | 1.00 | 0.88(0.51-1.52) | 1.23(0.70-2.17) | 0.74 (0.40-1.39) | 0.652 |
| BMI |  |  |  |  | *0.793^$^* |  |  |  |  |  | *0.529^$^* |
| <25 | 1.00 | 0.99(0.62-1.58) | 0.88(0.55-1.39) | 0.69(0.43-1.11) | 0.083 |  | 1.00 | 0.92(0.57-1.48) | 0.92(0.57-1.479) | 0.80(0.49-1.30) | 0.375 |
| ≥25 | 1.00 | 0.91(0.66-1.26) | 0.67(0.48-0.95)* | 0.61(0.42-0.90)* | 0.003 |  | 1.00 | 0.94(0.68-1.29) | 0.76(0.54-1.07) | 0.612(0.42-0.91)* | 0.008 |
| LDLc |  |  |  |  |  |  |  |  |  |  |  |
| Sex |  |  |  |  | *0.579^$^* |  |  |  |  |  | *0.560^$^* |
| Male | 1.00 | 0.76(0.56-1.03) | 1.06 (0.78-1.43) | 1.27(0.94-1.73) | 0.031 |  | 1.00 | 0.78(0.57-1.06) | 1.11(0.82-1.50) | 1.38(1.011-1.87)* | 0.008 |
| Female | 1.00 | 0.88(0.50-1.54) | 1.46(0.85-2.51) | 1.45(0.86-2.45) | 0.059 |  | 1.00 | 0.91 (0.50-1.65) | 1.40(0.80-2.47) | 1.58(0.92-2.74) | 0.038 |
| BMI |  |  |  |  | *0.580^$^* |  |  |  |  |  | *0.586^$^* |
| <25 | 1.00 | 0.70(0.45-1.08) | 1.28(0.85-1.93) | 1.23(0.81-1.88) | 0.078 |  | 1.00 | 0.75(0.48-1.18) | 1.33(0.88-2.03) | 1.48(0.96-2.27) | 0.014 |
| ≥25 | 1.00 | 0.81(0.57-1.15) | 1.04(0.74-1.46) | 1.34(0.95-1.89) | 0.031 |  | 1.00 | 0.80(0.56-1.13) | 1.05(0.75-1.48) | 1.39(0.99-1.96) | 0.017 |
| Non-HDLc | | | | | | | | | | | |
| Sex |  |  |  |  | *0.574^$^* |  |  |  |  |  | *0.714^$^* |
| Male | 1.00 | 1.15(0.85-1.57) | 1.12(0.82-1.52) | 1.39(1.01-1.91) | 0.063 |  | 1.00 | 1.25(0.92-1.71) | 1.28(0.94-1.74) | 1.54(1.12-2.12)** | 0.010 |
| Female | 1.00 | 0.85(0.47-1.48) | 1.38(0.80-2.37) | 1.27(0.74-2.19) | 0.139 |  | 1.00 | 0.80(0.44-1.46) | 1.50(0.85-2.64) | 1.33(0.76-2.33) | 0.088 |
| BMI |  |  |  |  | *0.463^$^* |  |  |  |  |  | *0.880^$^* |
| <25 | 1.00 | 1.012(0.66-1.56) | 1.31(0.86-2.00) | 1.23(0.79-1.91) | 0.214 |  | 1.00 | 1.06(0.69-1.66) | 1.46(0.95-2.26) | 1.30(0.82-2.04) | 0.127 |
| ≥25 | 1.00 | 1.10(0.77-1.56) | 1.11(0.78-1.57) | 1.44(1.02-2.04)* | 0.043 |  | 1.00 | 1.18(0.83-1.68) | 1.26(0.89-1.79) | 1.62(1.141-2.31)** | 0.007 |
| TC/HDLc | | | | | | | | | | | |
| Sex |  |  |  |  | *0.880^$^* |  |  |  |  |  | *0.454^$^* |
| Male | 1.00 | 0.97(0.71-1.3) | 1.24(0.91-1.70) | 1.41(1.02-1.94)* | 0.011 |  | 1.00 | 0.92(0.67-1.25) | 1.28(0.94-1.76) | 1.49(1.08-2.04)* | 0.002 |
| Female | 1.00 | 1.66(0.95-2.89) | 1.08(0.61-1.90) | 1.64(0.93-2.88) | 0.245 |  | 1.00 | 1.72(0.97-3.07) | 1.11(0.62-2.00) | 1.48(0.82-2.65) | 0.475 |
| BMI |  |  |  |  | *0.939^$^* |  |  |  |  |  | *0.807^$^* |
| <25 | 1.00 | 1.45(0.97-2.159) | 1.06(0.68-1.63) | 1.65(1.059-2.58)* | 0.109 |  | 1.00 | 1.472(0.99-2.22) | 1.07(0.69-1.68) | 1.62(1.03-2.56)* | 0.130 |
| ≥25 | 1.00 | 0.86(0.60-1.24) | 1.22(0.85-1.75) | 1.35(0.94-1.91) | 0.022 |  | 1.00 | 0.79(0.55-1.15) | 1.27(0.89-1.82) | 1.36(0.95-1.95) | 0.009 |
| LDLc/HDLc | | | | | | | | | | | |
| Sex |  |  |  |  | *0.922^$^* |  |  |  |  |  | *0.590^$^* |
| Male | 1.00 | 1.19(0.88-1.62) | 1.14(0.84-1.55) | 1.71(1.25-2.35)** | 0.002 |  | 1.00 | 1.12(0.83-1.52) | 1.120(0.88-1.63) | 1.72(1.26-2.36)** | 0.001 |
| Female | 1.00 | 1.34(0.77-2.32) | 1.06(0.60-1.88) | 1.92(1.07-3.42)* | 0.054 |  | 1.00 | 1.34(0.75-2.37) | 1.02(0.56-1.85) | 1.74(0.95-3.16) | 0.129 |
| BMI |  |  |  |  | *0.711^$^* |  |  |  |  |  | *0.668^$^* |
| <25 | 1.00 | 1.16(0.78-1.73) | 0.93(0.60-1.46) | 1.79(1.14-2.80)* | 0.043 |  | 1.00 | 1.22(0.81-1.84) | 0.98(0.62-1.54) | 1.91(1.20-3.02)** | 0.024 |
| ≥25 | 1.00 | 1.21(0.84-1.74) | 1.22(0.86-1.73) | 1.74(1.23-2.47)** | 0.002 |  | 1.00 | 1.08(0.75-1.55) | 1.25(0.88-1.78) | 1.65(1.16-2.35)** | 0.003 |
| TG/HDLc | | | | | | | | | | | |
| Sex |  |  |  |  | *0.633^$^* |  |  |  |  |  | *0.233^$^* |
| Male | 1.00 | 1.06(0.78-1.46) | 1.09(0.789-1.51) | 1.14(0.82-1.58) | 0.440 |  | 1.00 | 0.94(0.69-1.29) | 1.05(0.76-1.46) | 1.14(0.82-1.59) | 0.390 |
| Female | 1.00 | 0.77(0.44-1.35) | 0.88(0.50-1.55) | 0.84(0.48-1.48) | 0.704 |  | 1.00 | 0.90(0.50-1.61) | 0.82(0.45-1.48) | 0.71(0.39-1.29) | 0.239 |
| BMI |  |  |  |  | *0.981^$^* |  |  |  |  |  | *0.158^$^* |
| <25 | 1.00 | 0.73(0.49-1.10) | 0.96(0.63-1.48) | 0.98(0.63-1.55) | 0.809 |  | 1.00 | 0.76(0.50-1.14) | 0.82(0.53-1.28) | 0.83(0.52-1.33) | 0.492 |
| ≥25 | 1.00 | 1.22(0.83-1.80) | 1.12(0.77-1.64) | 1.17(0.81-1.71) | 0.599 |  | 1.00 | 1.08(0.73-1.59) | 1.16(0.79-1.69) | 1.21(0.83-1.76) | 0.299 |

Compared with the lowest quarter, **P*<0.05,***P*≤0.01,*** *P*≤0.001; *^$^P*value for interaction.
